# Supplementary figures and images for: Biochemical characterization of a GDP-mannose transporter from Chaetomium thermophilum
Source: PLoS One. 2023 Apr 20;18(4):e0280975. doi: 10.1371/journal.pone.0280975 (PMC10118193; doi:10.1371/journal.pone.0280975)

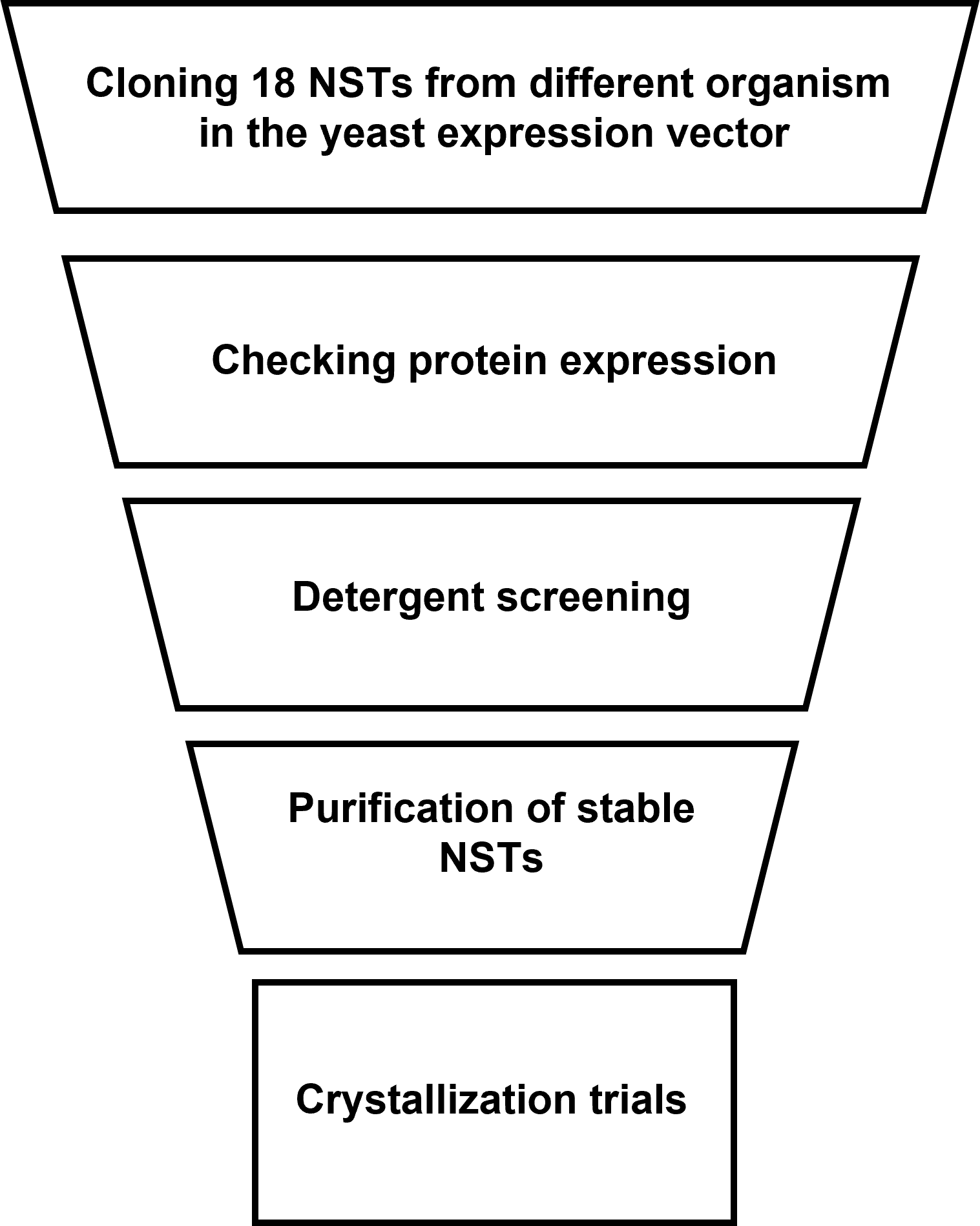

Supplement: S1 Fig — (TIF) [file pone.0280975.s001.tif]

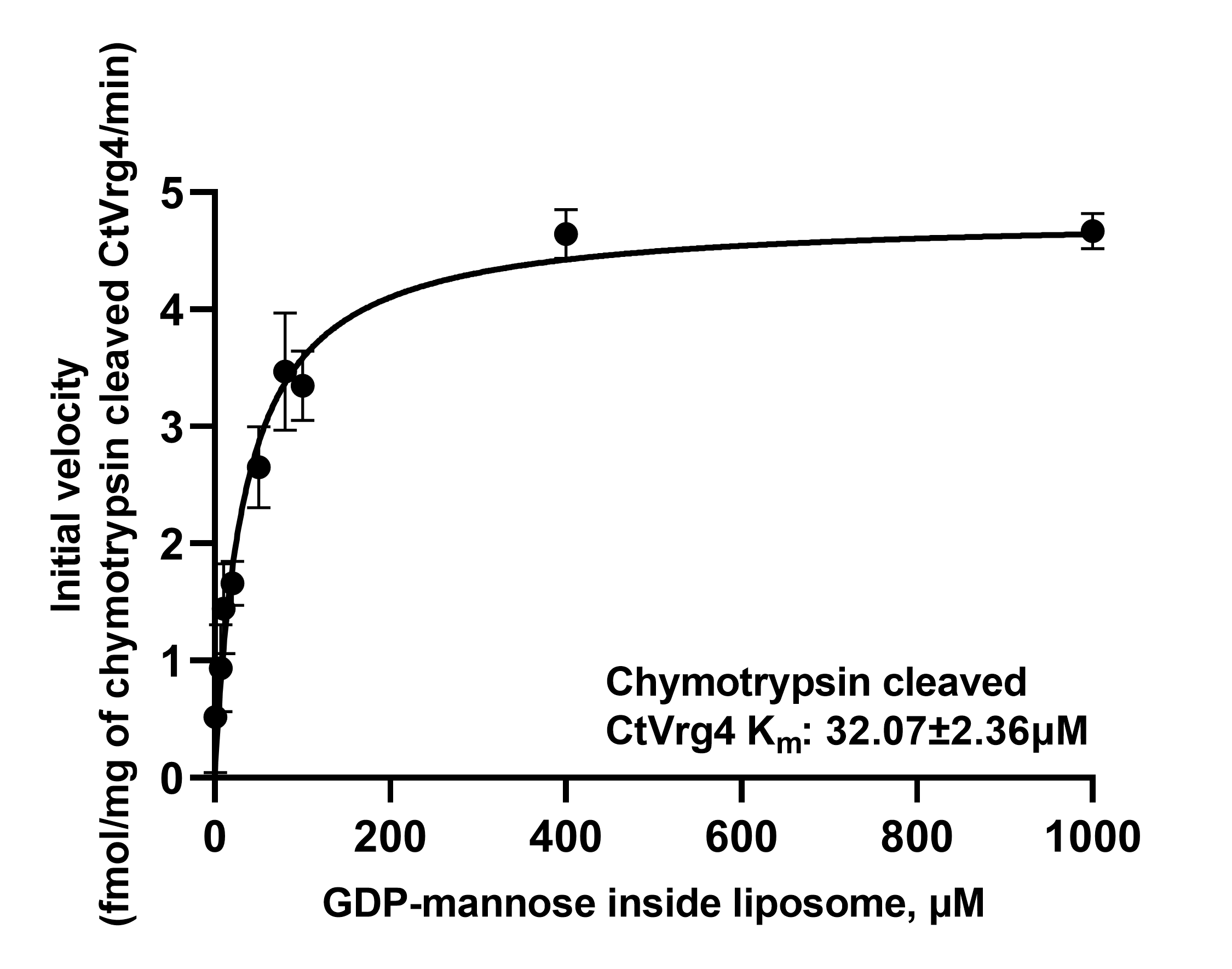

Supplement: S2 Fig — Values are the means of three independent biological repeats (each done in technical triplicate). Errors are indicated as SD. Km was calculated by non-linear fit using the GraphPad Prism software. (TIF) [file pone.0280975.s002.tif]

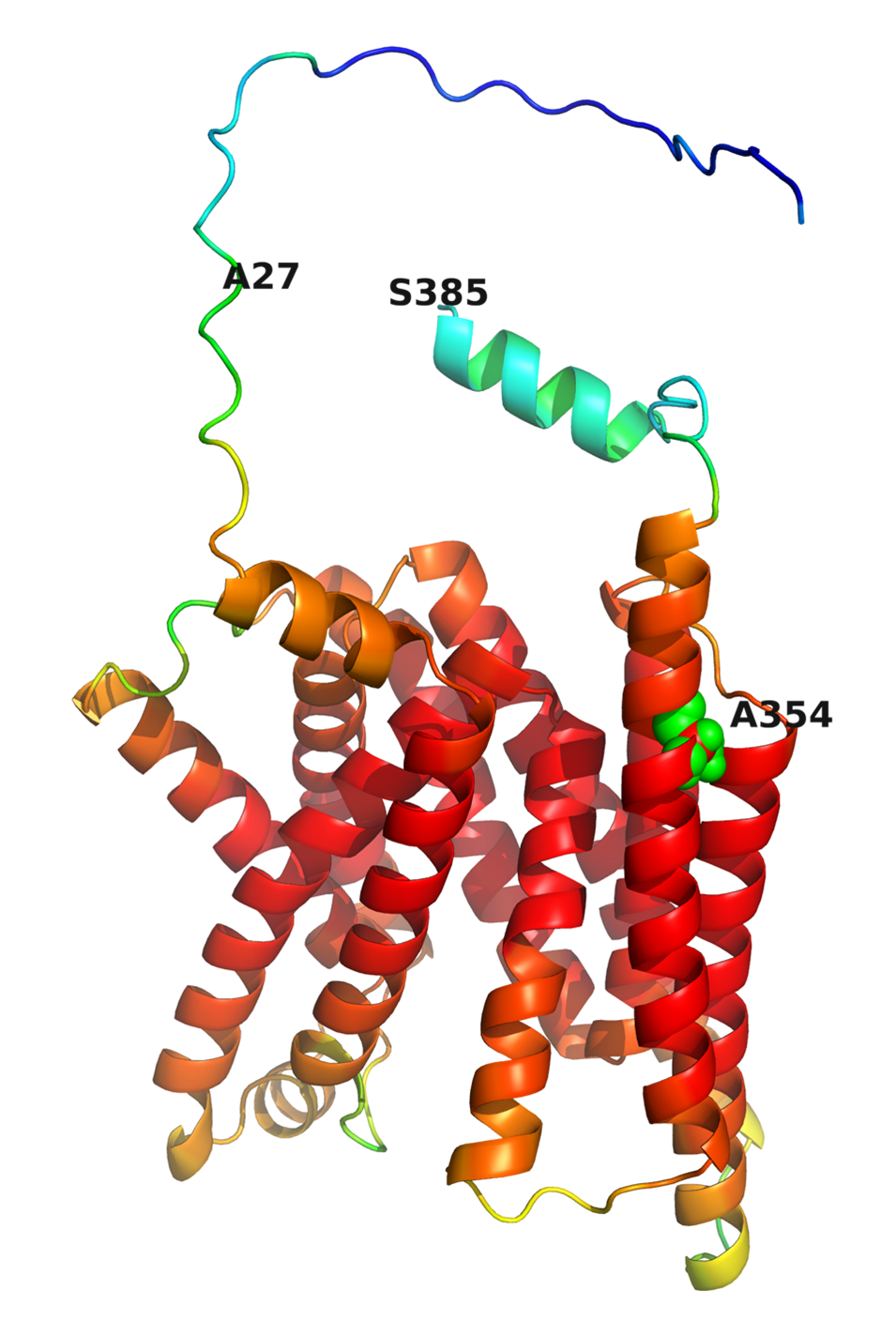

Supplement: S3 Fig — Colors represent the predicted confidence value of the structure. The confidence value decreases as the color changes from red to blue. The figure shows that the transmembrane regions are predicted with high confidence–in red. The N-terminus region is floppy and is predicted poorly. Note that the C-terminal helix is predicted with intermediate confidence (green). Amino acid Alanine 27 (A27) and the C-terminal residue (S385) are labeled for reference. A354, is the last residue in the CtVrg4Δ31 construct. (TIF) [file pone.0280975.s003.tif]

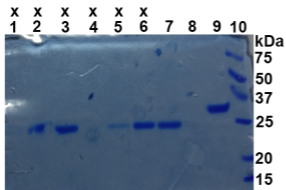

Lane -

1 - Empty

2 to 7 - Chymotrypsin cleaved CtVrg4

8 - Empty

9 - Full length CtVrg4

10 - Molecular weight marker

Supplement: S1 Raw image — (PDF) [file pone.0280975.s006.pdf]
