# Supplementary material for: Biochemical characterization of a GDP-mannose transporter from Chaetomium thermophilum
Source: PLoS One. 2023 Apr 20;18(4):e0280975. doi: 10.1371/journal.pone.0280975 (PMC10118193; doi:10.1371/journal.pone.0280975)
Supplement: S1 Table — (DOCX) [file pone.0280975.s004.docx]

**Supplementary Information**

**Biochemical characterization of a GDP-mannose transporter from *Chaetomium thermophilum***

Gowtham Thambra Rajan Premageetha^1,2,3^, KanagaVijayan Dhanabalan^1,2^, Sucharita Bose^2,#^, Lavanyaa Manjunath^2,#^, Deepthi Joseph^2,#^, Aviv Paz^4^, Samuel Grandfield^4^, Vinod Nayak^2,#^, Luis M.Bredeston^5^, Jeff Abramson^4^ and Subramanian Ramaswamy^1,2,*^.

^1^Biological Sciences, Purdue University, West Lafayette, Indiana, 47907 , USA.

^2^Institute for Stem Cell Science and Regenerative Medicine, Bengaluru, Karnataka, 560065, India.

^3^Manipal Academy of Higher Education, Tiger Circle Road, Manipal, Karnataka, 576104, India.

^4^Department of Physiology, David Geffen School of Medicine at UCLA, Los Angeles, CA 90096, USA.

^5^Departamento de Química Biológica-IQUIFIB, Facultad de Farmacia y Bioquímica, Universidad de Buenos Aires-CONICET, Ciudad Autónoma de Buenos Aires, Junín 956 (1113), Argentina.

^#^Authors conducted their work for this manuscript in the associated affiliation.

* corresponding author

E-mail: [subram68@purdue.edu](mailto:subram68@purdue.edu)

**Table S1. NSTs identified for crystallization through homology search.**

| **Gene Name** | **Species** | **Substrate** |
| --- | --- | --- |
| SLC35A3 | *Xenopus tropicalis* | UDP-GlcNAc |
| SLC35A3 | *Mus musculus* | UDP-GlcNAc |
| SLC35A3 | *Caenorhabditis elegans* | UDP-GlcNAc |
| SLC35A2 | *Schizosaccharomyces pombe* | UDP-Gal |
| SRF-3 | *Caenorhabditis elegans* | UDP-Gal/  UDP-GlcNAc |
| SQV-7 | *Caenorhabditis elegans* | UDP-Gal/  UDP-GlcNAc/  UDP-GlcA, |
| CeZK896.6/CeNSTP-5/NM_001268750.1 | *Caenorhabditis elegans* | Unknown |
| SLC35C1 | *Homo sapiens* | GDP-Fucose |
| SLC35A1 | *Mus musculus* | CMP-Sia |
| GlNST-1/XM_001706177.1 | *Giardia lamblia* | UDPGlcNAc |
| EhNST-1/ XM_643503.2 | *Entamoeba hystolytica* | UDP-Gal |
| SpNST-UDPglcNac/  NM_001021334.2 | *Schizosaccharomyces pombe* | UDP-GlcNAc (predicted) |
| CeNSTP-8  /NM_071203.2 | *Caenorhabditis elegans* | Unknown |
| SLC35F6 | *Homo sapiens* | Unknown |
| SLC35E2 | *Homo sapiens* | Unknown |
| SLC35A1 | *Homo sapiens* | CMP-Sia |
| SLC35E4 | *Homo sapiens* | Unknown |
| SLC35A5 | *Homo sapiens* | Unknown |
| SLC35F1 | *Homo sapiens* | Unknown |
| SLC35B1 | *Homo sapiens* | ATP/ADP |
| SLC35F2 | *Homo sapiens* | Unknown |
| SLC35F5 | *Homo sapiens* | Unknown |
| SLC35C2 | *Homo sapiens* | Unknown |
| SLC35D1 | *Homo sapiens* | UDP-Sugars/UMP |
| SLC35G1 | *Homo sapiens* | Unknown |
| SLC35D2 | *Homo sapiens* | UDP-GlcNAc/UDP-GalNAc |
| GDP-mannose transporter | *Chaetomium thermophilum* | GDP-Mannose/GMP |
