# Supplementary material for: Biochemical characterization of a GDP-mannose transporter from Chaetomium thermophilum
Source: PLoS One. 2023 Apr 20;18(4):e0280975. doi: 10.1371/journal.pone.0280975 (PMC10118193; doi:10.1371/journal.pone.0280975)
Supplement: S2 Table — (DOCX) [file pone.0280975.s005.docx]

**Supplementary Information**

**Biochemical characterization of a GDP-mannose transporter from *Chaetomium thermophilum***

Gowtham Thambra Rajan Premageetha^1,2,3^, KanagaVijayan Dhanabalan^1,2^, Sucharita Bose^2,#^, Lavanyaa Manjunath^2,#^, Deepthi Joseph^2,#^, Aviv Paz^4^, Samuel Grandfield^4^, Vinod Nayak^2,#^, Luis M.Bredeston^5^, Jeff Abramson^4^ and Subramanian Ramaswamy^1,2,*^.

^1^Biological Sciences, Purdue University, West Lafayette, Indiana, 47907 , USA.

^2^Institute for Stem Cell Science and Regenerative Medicine, Bengaluru, Karnataka, 560065, India.

^3^Manipal Academy of Higher Education, Tiger Circle Road, Manipal, Karnataka, 576104, India.

^4^Department of Physiology, David Geffen School of Medicine at UCLA, Los Angeles, CA 90096, USA.

^5^Departamento de Química Biológica-IQUIFIB, Facultad de Farmacia y Bioquímica, Universidad de Buenos Aires-CONICET, Ciudad Autónoma de Buenos Aires, Junín 956 (1113), Argentina.

^#^Authors conducted their work for this manuscript in the associated affiliation.

* corresponding author

E-mail: [subram68@purdue.edu](mailto:subram68@purdue.edu)

**Table S2. Percentage sequence identity of known and putative GDP-mannose transporters from different species of fungi - alignment carried out using Clustal Omega.**

|  | ***S. cerevisiae*** | ***C. albicans*** | ***C. glabrata*** | ***S. pombe*** | ***A.***  ***niger*** | ***N. crassa*** | ***N. fumigata*** | ***K. pastoris*** | ***C. globosum*** |
| --- | --- | --- | --- | --- | --- | --- | --- | --- | --- |
| ***C. thermophilum*** | 53.61 | 54.15 | 55.94 | 59.53 | 61.56 | 77.75 | 61.29 | 56.31 | 81.25 |
| ***S. cerevisiae*** |  | 64.33 | 75.31 | 58.18 | 56.5 | 54.19 | 57.83 | 63.44 | 54.03 |
| ***C. albicans*** |  |  | 60.75 | 61.42 | 60.28 | 55.37 | 61.52 | 71.04 | 52.11 |
| ***C. glabrata*** |  |  |  | 55.95 | 54.66 | 53.87 | 55.73 | 59.74 | 55.11 |
| ***S. pombe*** |  |  |  |  | 65.6 | 58.82 | 67.06 | 61.42 | 58.65 |
| ***A. niger*** |  |  |  |  |  | 60.9 | 87.4 | 59.94 | 60.21 |
| ***N. crassa*** |  |  |  |  |  |  | 58.89 | 55.96 | 76.41 |
| ***N. fumigata*** |  |  |  |  |  |  |  | 61.28 | 59.15 |
| ***K. pastoris*** |  |  |  |  |  |  |  |  | 55.79 |
